# N-myc downstream-regulated gene 1 promotes oxaliplatin-triggered apoptosis in colorectal cancer cells via enhancing the ubiquitination of Bcl-2

**DOI:** 10.18632/oncotarget.17711

**Published:** 2017-05-09

**Authors:** Xiao Yang, Fan Zhu, Chaoran Yu, Jiaoyang Lu, Luyang Zhang, Yanfeng Lv, Jing Sun, Minhua Zheng

**Affiliations:** ^1^ Department of General Surgery, Ruijin Hospital, Shanghai Jiao Tong University, School of Medicine, Shanghai 200025, P.R.China; ^2^ Shanghai Minimally Invasive Surgery Center, Ruijin Hospital, Shanghai Jiao Tong University, School of Medicine, Shanghai 200025, P.R.China; ^3^ Department of Colorectal Surgery, Second Hospital of Shandong University, Shandong University, Jinan 250000, P.R.China

**Keywords:** apoptosis, colorectal cancer, neoadjuvant chemotherapy, NDRG1, Bcl-2

## Abstract

N-myc downstream-regulated gene1 (*NDRG1*) has been identified as a potent tumor suppressor gene. The molecular mechanisms of anti-tumor activity of NDRG1 involve its suppressive effects on a variety of tumorigenic signaling pathways. The purpose of this study was to investigate the role of NDRG1 in the apoptosis of colorectal cancer (CRC) cells. We first collected the clinical data of locally advanced rectal cancer (LARC) patients receiving oxaliplatin-based neoadjuvant chemotherapy in our medical center. Correlation analysis revealed that NDRG1 positively associated with the downstaging rates and prognosis of patients. Then, the effects of over-expression and depletion of *NDRG1* gene on apoptosis of colorectal cancer were tested *in vitro* and *in vivo*. *NDRG1* over-expression promoted apoptosis in colorectal cancer cells whereas depletion of *NDRG1* resulted in resistance to oxaliplatin treatment. Furthermore, we observed that Bcl-2, a major anti-apoptotic protein, was regulated by NDRG1 at post-transcriptional level. By binding Protein kinase Cα (PKCα), a classical regulating factor of Bcl-2, NDRG1 enhanced the ubiquitination and degradation of Bcl-2, thus promoting apoptosis in CRC cells. In addition, NDRG1 inhibited tumor growth and promoted apoptosis in mouse xenograft model. In conclusion, NDRG1 promotes oxaliplatin-triggered apoptosis in colorectal cancer. Therefore, colorectal cancer patients can be stratified by the expression level of NDRG1. NDRG1-positive patients may benefit from oxaliplatin-containing chemotherapy regimens whereas those with negative NDRG1 expression should avoid the usage of this cytotoxic drug.

## INTRODUCTION

Preoperative chemoradiation therapy (CRT) followed by total mesorectal excision (TME) has been currently considered as the standard treatment strategy for locally advanced rectal cancer (LARC) [1–3]. However, the optimal type of neoadjuvant therapy regimen is still unclarified. Several randomized trials comparing the efficacy and toxicities of CRT with or without oxaliplatin (L-OHP), as the preoperative treatment for locally advanced rectal cancer have been reported [4–7]. Although the results were inconsistent, oxaliplatin was still a reasonable candidate for inclusion into neoadjuvant chemotherapy regimens, which was further evidenced by studies demonstrating that oxaliplatin was able to induce apoptotic cell death in colorectal cancer (CRC) cells [8, 9]. However, the mechanisms by which oxaliplatin trigger death signal in cancer cells have not been fully defined yet.

Apoptosis is a morphologically and molecularly distinct form of programmed cell death. This conserved cellular suicide program is initiated either ‘intrinsically’[10, 11] through cellular stress that impinge on the mitochondria or ‘extrinsically’[12, 13] from extracellular ligands that activate death receptors at the cytomembrane. One of the most important advances in cancer research is the recognition that apoptosis is crucially involved in the regulation of tumor formation and also critically determines chemotherapy treatment response [12, 14, 15]. Thus, understanding of the molecular events that regulate apoptosis in response to anticancer chemotherapy, and how cancer cells evade apoptotic death provides novel insights for a more rational approach to develop molecular targeted therapies for combating cancer.

N-myc downstream-regulated gene 1 (*NDRG1*) plays a potent role in modulating metastatic ability in multiple types of solid tumor including CRC [16, 17], breast cancer [18, 19] and prostate cancer [20, 21]. Also, NDRG1 has been linked to many cellular processes including cell cycle, apoptosis, and cellular differentiation [22–26]. Plenty of studies indicate that over-expression of *NDRG1* is able to inhibit the invasion and metastasis of CRC. The molecular mechanism this anti-tumor activity involves its suppressive effects on several tumorigenic signaling pathways such as Wnt [27, 28], phosphatidylinositol 3-kinase (PI3K) [29, 30] and transforming growth factor-β (TGF-β) [31] pathway. Moreover, our previous work revealed a significant inverse correlation of NDRG1 expression with tumor stage, differentiation status and metastasis in CRC patients, suggesting the clinical significance of *NDRG1*[32]. To date, however, the underlying mechanism by which *NDRG1* affects apoptosis has not been fully elucidated. Hence, the specific functions of *NDRG1* in apoptosis need to be further investigated.

## RESULTS

### NDRG1 positively correlated to the downstaging rate and prognosis of LARC patients receiving neoadjuvant chemotherapy

**Figure 1:**
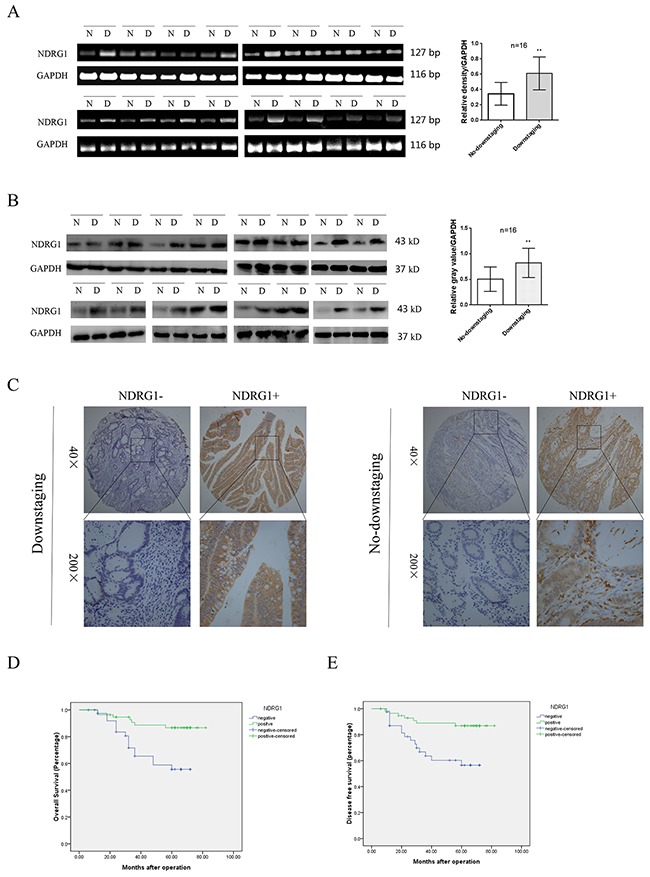
Expression of NDRG1 and its clinical significance in LARC patients **(A)** RT-PCR showing *NDRG1* mRNA levels in 16 selected samples from downstaging and no-downstaging group respectively. Samples were selected by random numbers generated with SAS software. **(B)** Western blot analysis of NDRG1 expression in selected CRC samples. **(C)** NDRG1 protein levels in tissues from patients of downstaging and no-downstaging groups were measured with IHC staining. **(D, E)** CRC patients with negative expression of NDRG1 presented poorer overall survival (D) and disease free survival (E) compared with those with positive expression of NDRG1. Bars indicated SD. *, *P* < 0.05, **, *P* < 0.01.

A total of 97 LARC patients were enrolled into our study. Colonoscopy was performed on each patient to validate the diagnosis. After 2 cycles of chemotherapy and subsequent surgical resection, patients were divided into downstaging or no-downstaging group according to the pathological staging results. Sixteen patients were randomly selected in each group. To evaluate the potential effect of NDRG1 on the efficacy of oxaliplatin-based chemotherapy, total RNA was extracted from cancer tissues and subjected to reverse transcription-polymerase chain reaction (RT-PCR). As shown, *NDRG1* transcript levels were significantly higher in cancer tissues from downstaging patients relative to those from no-downstaging ones (Figure 1A). Moreover, higher expression of NDRG1 in downstaging group was further confirmed at the protein level by western blots (Figure 1B). A tissue microarray containing specimens from all enrolled patients was analyzed by immunohistochemistry (IHC) (Figure 1C). In downstaging group, the positive expression of NDRG1 was detected in 39 cases (67.2%), whereas only 18 cases (46.1%) showed positive signal in no-downstaging group (*R* = 0.210, *P* = 0.039, Table 1). The detailed clinical information of each patient was listed in supplementary Table 1.

**Table 1:** Relationship between NDRG1 expression and response to neoadjuvant chemotherapy

	Case	Response to chemotherapy		*P* value
	n=97	Downstaging	No-downstaging	
NDRG1-positive	57	39	18	0.039
NDRG1-negative	40	19	21	

To further investigate the clinical significance of NDRG1 in CRC, we analyzed the possible correlation between NDRG1 expression and clinicopathologic variables (Table 2). Our data showed a significant inverse correlation between NDRG1 and tumor size (*R* = -0.355, *P* = 0.001), local invasion (*R* = -0.318, *P* = 0.002), lymphatic invasion (*R* = -0.287, *P* = 0.005) and TNM stage (*R* = -0.287, *P* = 0.005). Moreover, Kaplan–Meier analysis revealed that patients in the NDRG1 negative group (n = 40) had a significantly poorer overall survival than those in the NDRG1 positive group (n = 57; log-rank = 10.37; *P* = 0.001; Figure 1D). Additionally, NDRG1 positive expression was associated with a longer disease-free survival time (log-rank = 9.974, *P* = 0.002, Figure 1E). These findings suggested that NDRG1 positively correlated to the downstaging rate of LARC patients receiving neoadjuvant chemotherapy. Moreover, NDRG1 might function as a potential prognostic marker.

**Table 2:** Relationship between NDRG1 and clinicopathologic variables

Variable	Case	NDRG1 expression		*P* value
		Positive	Negative	
	n=97	n=57	n=40	
**Age (years)**				0.520
≤65	52	29	23	
>65	45	28	17	
**Gender**				0.119
Male	72	39	33	
Female	25	18	7	
**ECOG performance**				0.183
0	68	37	31	
1	29	20	9	
**Tumor size (cm)**				0.001
≤4 ×3	52	39	13	
>4 ×3	45	18	27	
**Location from anal verge**				0.290
0-5cm	52	28	24	
5-10cm	45	29	16	
**Local invasion**				0.002
T2	26	22	4	
T3+T4	71	35	36	
**Lymphatic invasion**				0.005
N0	27	22	5	
N1+N2	70	35	35	
**TNM stage**				0.005
II	27	22	5	
III	70	35	35	

The staging results were determined by two independent pathologists according to the 7th edition of the AJCC Cancer Staging system.

### NDRG1 was necessary for oxaliplatin-triggered apoptosis

**Figure 2:**
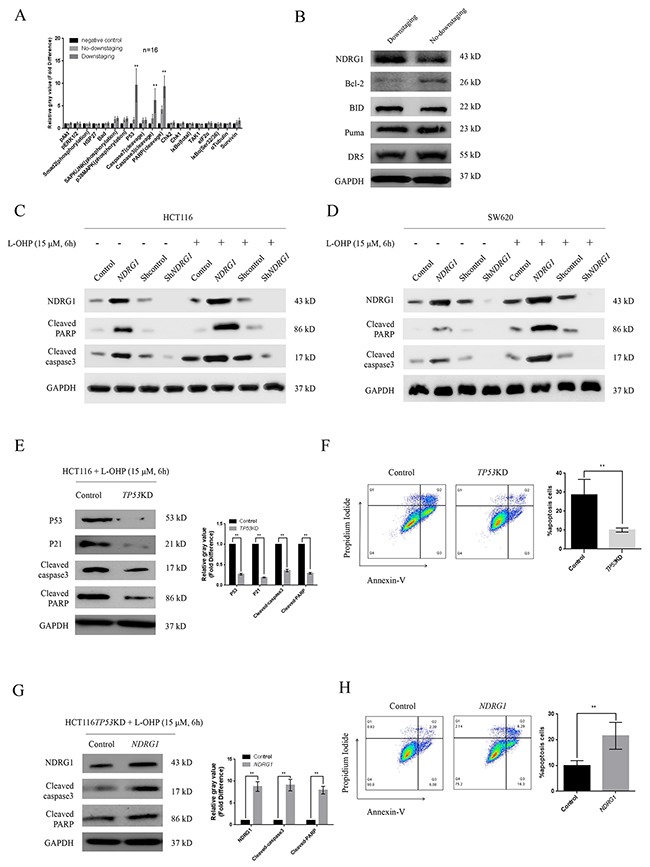
NDRG1 was necessary for oxaliplatin-triggered apoptosis **(A)** Pathscan array showing elevated protein levels of P53, cleaved-caspase-3, and cleaved-PARP in samples from downstaging group. **(B)** Representative western blots of several main apoptosis-regulating proteins, NDRG1 expression was increased while Bcl-2 was degraded in tissues from downstaging group. **(C, D)** Western blot assay showing protein levels of cleaved-PARP and –caspase-3 of HCT116 (C) and SW620 (D) cells and their *NDRG1* derivates with or without oxaliplatin treatment. **(E, F)** *TP53* knockdown attenuated oxaliplatin-triggered apoptosis. Apoptosis in HCT116*TP53KD* and control cells were measured with western blots (E) and confirmed by flow cytometry with Annexin V/PI (F). P21 was used as an evidence of successful knockdown of *TP53*. **(G, H)** *NDRG1* expression rescued the drug resistance induced by *TP53* knockdown. Apoptosis was measured with western blots (G) and flow cytometry assay (H). All histograms showed mean values from three independent experiments; bars indicated SD. *, *P* < 0.05, **, *P* < 0.01.

To gain insight into the mechanisms of significant tumor regression in NDRG1-positive patients, we detected altered activities in several apoptotic pathways via a pathscan array using protein extracted from selected specimens. As shown, after two cycles of chemotherapy, the protein levels of P53, cleaved-caspase-3, and cleaved-PARP were dramatically elevated in downstaging group (Figure 2A), indicating that the remarkable tumor regression in downstaging patients was due to substantial apoptosis induced by oxaliplatin. Western blot was then performed to analyze several apoptosis regulating factors. Intriguingly, upon the increase of NDRG1, a loss of Bcl-2 protein was detected in specimens from downstaging group while protein levels of other molecules stayed stable (Figure 2B and Supplementary Figure 1A), suggesting NDRG1 might promote apoptosis via targeting Bcl-2, a classical potent apoptosis suppressor. To investigate the role of NDRG1 in the apoptotic procedure, we generated HCT116*NDRG1* cells stably expressing human *NDRG1* and HCT116sh*NDRG1* cells depleted of *NDRG1*. The magnitude of apoptosis was measured with western blots. As shown, significant higher levels of cleaved-caspase-3 and cleaved-PARP were detected in HCT116*NDRG1* cells (Figure 2C). In contrast, silencing of *NDRG1* resulted in remarkable blockage of apoptosis (Figure 2C). Similar results were also observed in SW620 cells (Figure 2D). Moreover, the apoptosis induced by *NDRG1* was potentiated by oxaliplatin. After oxaliplatin treatment, the protein levels of cleaved-caspase-3 and -PARP in HCT116- and SW620*NDRG1* cells were drastically elevated compared to those untreated whereas control cells showed moderate elevation (Figure 2C, 2D and Supplementary Figure 1B, 1C). Additionally, sh*NDRG1* cells showed negligible signal of cleaved-caspase-3 and -PARP even after drug treatment (Figure 2C, 2D and Supplementary Figure 1B, 1C). These results suggested that *NDRG1* could increase the sensitivity of CRC cells to oxaliplatin via promoting apoptosis.

Previous study reported that NDRG1 is necessary to P53-induced apoptosis. To further validate the effect of NDRG1, a selective knockdown of *TP53* gene was performed in HCT116 cells (*TP53*KD), leading to attenuated response to oxaliplatin (Figure 2E, 2F). However, ectopic *NDRG1* expression could restore the sensitivity of *TP53*KD cells to oxaliplatin (Figure 2G, 2H). The results in HT29 cells, a *TP53* defective and oxaliplatin-resistant CRC cell line, were similar in the same experiment setting (Supplementary Figure 2A, 2B). Taken together, these data indicated that oxaliplatin-triggered apoptosis depends on *NDRG1* instead of *TP53*.

### NDRG1 induced the degradation of Bcl-2 via ubiquitin-proteasome pathway

**Figure 3:**
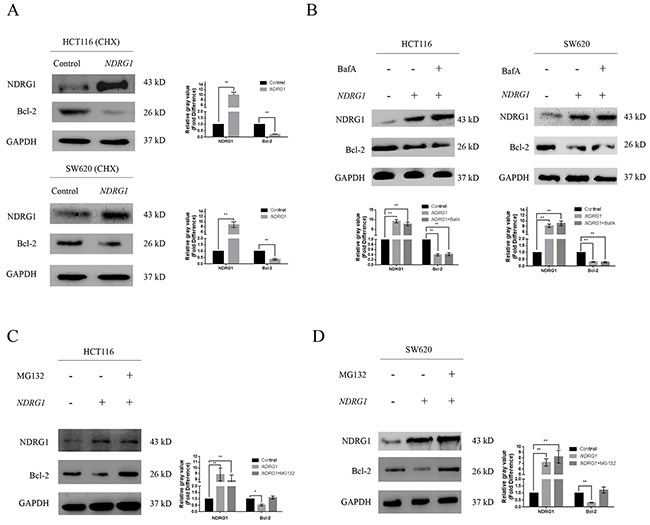
NDRG1 induced degradation of Bcl-2 via ubiquitin-proteasome pathway **(A)** HCT116 and SW620 cells pretreated with with CHX (20μM, 6h), a classical protein synthesis inhibitor, were transfected with *NDRG1*. Protein levels of Bcl-2 were analyzed with western blots 24 hours later. **(B)** After treated with lysosomal inhibitor BafilomycinA1 (BafA, 0.2μM), protein levels of Bcl-2 were analyzed in control and *NDRG1*-transfected cells. **(C, D)** Western blots showing accumulation of Bcl-2 protein after treatment with MG132 (10μM) in *NDRG1*-transfected cells. All histograms showed mean values from three independent experiments; bars indicated SD. *, *P* < 0.05, **, *P* < 0.01.

**Figure 4:**
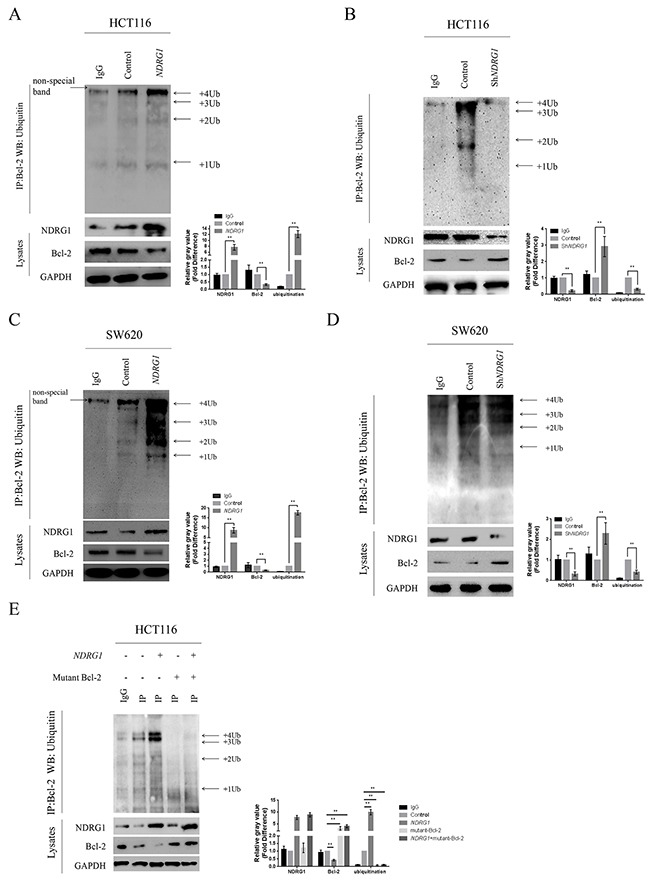
NDRG1 enhanced Bcl-2 ubiquitination **(A)** Western blots with anti-ubiquitin antibody revealed Bcl-2 ubiquitination level in immunoprecipitates prepared from HCT116 cells over-expressing NDRG1 was much higher than control cells. The bands of the various ubiquitylated forms of Bcl-2 were as indicated. **(B)** Knockdown of *NDRG1* suppressed Bcl-2 ubiquitinaiton. **(C, D)** Bcl-2 ubiquitination levels in SW620*NDRG1* (C) or -sh*NDRG1* (D) cells. **(E)** the loss of BH4 domain impaired the ubiquitination of Bcl-2. HCT116*NDRG1* cells were transfected with mutant (BH4 deletion) *Bcl-2*. Immunoprecipitation assay was then performed to show different levels of ubiquitination. All histograms showed mean values from three independent experiments; bars indicated SD. *, *P* < 0.05, **, *P* < 0.01.

By blocking polymerization of Bax, Bcl-2 suppresses apoptosis and contributes to drug resistance in cancer cells. We first confirmed the counteractive role of Bcl-2 in oxaliplatin-triggered apoptosis in CRC cells. HCT116 cells were transfected with high amount of *Bcl-2* constructs (10μg), and apoptosis was dramatically attenuated (Supplementary Figure 2C). In addition, the over-expression of *Bcl-2* also suppressed the magnitude of apoptosis in HCT116*NDRG1* cells (Supplementary Figure 2D), further validated our above-mentioned hypothesis that NDRG1 might promote apoptosis by targeting Bcl-2. Next, to explore the mechanisms for the loss of Bcl-2 protein, we treated HCT116 and SW620 cells with cycloheximide (CHX), a translation inhibitor, before *NDRG1* ectopic expression. However, Bcl-2 protein level still underwent a fast decline upon *NDRG1* expression (Figure 3A), which indicated the loss of Bcl-2 was due to enhanced degradation instead of inhibition of protein synthesis. We then investigated if Bcl-2 degradation could be modulated by NDRG1. The pathway through which Bcl-2 degraded was investigated using both lysosome and proteasome pathway inhibitors. As shown, the lysosomal inhibitor BafilomycinA1 (BafA) had no effect on Bcl-2 degradation (Figure 3B) while the proteasomal inhibitor MG132 reversed the degradation of Bcl-2 in *NDRG1* cells (Figure 3C, 3D). Moreover, proteins degraded by proteasome system are often first conjugated to multiple copies of ubiquitin through covalent binding. To verify the role of NDRG1 in Bcl-2 ubiquitination, immunoprecipitation assay was performed in *NDRG1* over-expression and knockdown cell models. As shown, *NDRG1* over-expressed cells displayed a much higher Bcl-2 ubiquitination level than control cells (Figure 4A, 4C) whereas sh*NDRG1* cells demonstrated the opposite (Figure 4B, 4D).

Bcl-2 contains 4 conserved homology domains (BH1–4), among which BH4 containing lysine residues is very crucial to the process of ubiquitination. To further confirm our results, the BH4 deletion mutant of *Bcl-2* were constructed and introduced into HCT116 cells. As expected, the ubiquitination of Bcl-2 was significantly decreased in mutant cells even after *NDRG1* over-expression (Figure 4E). These results suggested that NDRG1 induced the degradation of Bcl-2 via ubiquitin-proteasome pathway. The decrease of Bcl-2 subsequently resulted in enhanced apoptosis.

### NDRG1 enhanced Bcl-2 ubiquitination by binding PKCα

**Figure 5:**
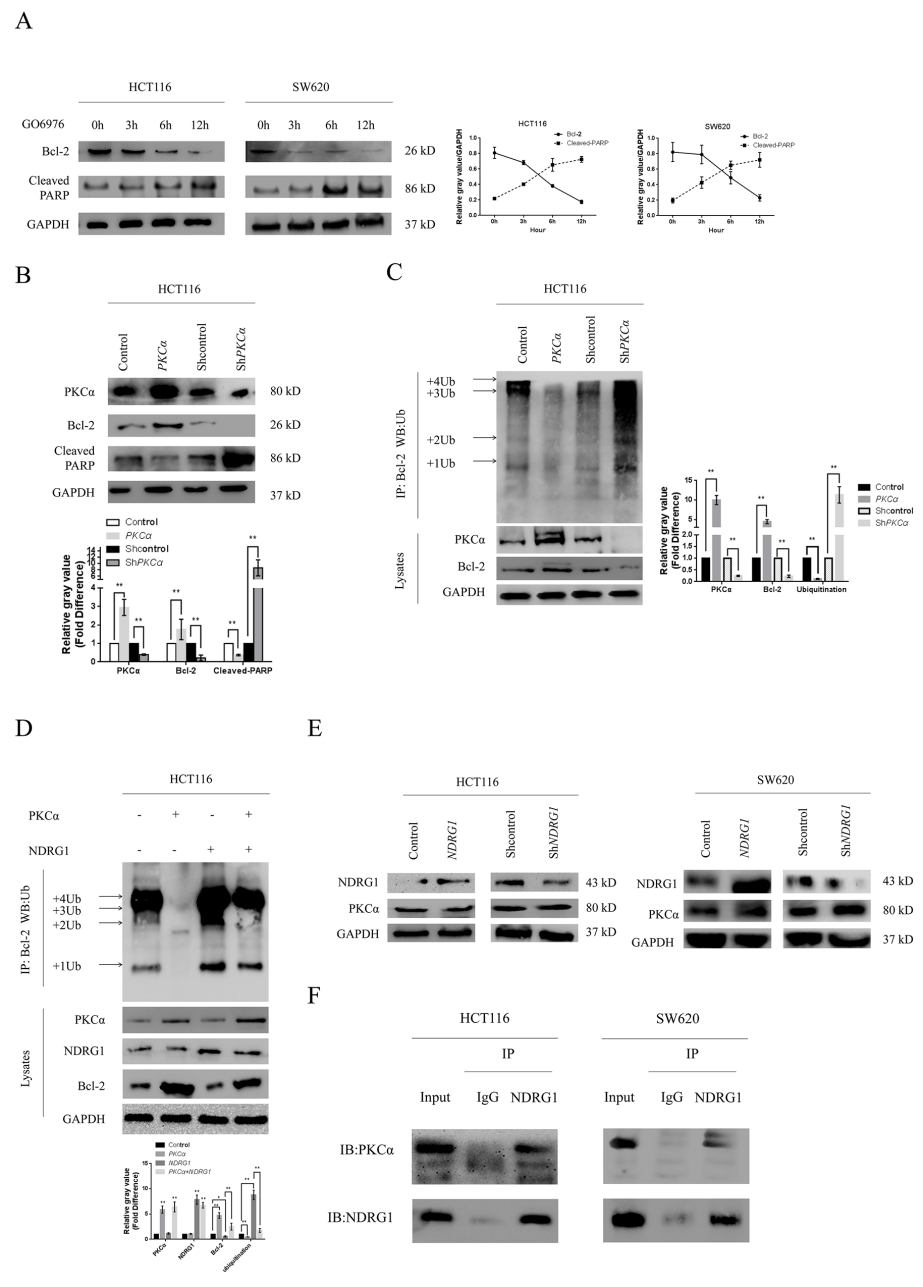
NDRG1 enhanced Bcl-2 ubiquitination via binding PKCα **(A)** Bcl-2 was fast degraded with the treatment of GO6976, an inhibitor of PKC, accompanied with induction of apoptosis. Cleaved-PARP was a visualized evidence of cell apoptotic death. **(B)** HCT116 cells were transfected with pcDNA3.1/*PKCα* vector or pLKO/sh*PKCα* vector (10μg). Then protein levels of Bcl-2 and cleaved-PARP were measured with western blots. **(C)** Western blots showing different Bcl-2 ubiquitination levels in HCT116 derivates with altered *PKCα* status. **(D)** NDRG1 reversed the suppression of Bcl-2 ubiquitination induced by *PKCα* over-expression. Immunoprecipation assay was performed as above described. **(E)** NDRG1 was unable to modulate protein level of PKCα. Protein levels in different NDRG1 derivates of HCT116 cells were detected by western blots. **(F)** NDRG1 directly interacted with PKCα in CRC cell lines. All histograms showed mean values from three independent experiments; bars indicated SD. *, *P* < 0.05, **, *P* < 0.01.

Previous studies demonstrated that Bcl-2 underwent ubiquitin-proteasome pathway after inhibition of protein kinase C (PKC), accompanied with induction of apoptosis [33]. According to the antagonism relationship between NDRG1 and PKC family in hematologic malignancy [26], we assumed that similar mechanisms existed in CRC. To verify this hypothesis, we treated HCT116 and SW620 cells with GO6976 (10μM), a specific inhibitor of the PKCα and PKCβ. As expected, Bcl-2 degraded in a time-dependent manner (Figure 5A). We then investigated the consequences of over-expression and depletion of *PKCα* and *PKCβ* respectively. As shown, over-expression of *PKCα* stabilized Bcl-2 and suppressed magnitude of apoptosis whereas depletion of *PKCα* resulted in the opposite (Figure 5B). However, neither over-expression nor knockdown of *PKCβ* affected Bcl-2 or apoptosis (Supplementary Figure 2E). Immunoprecipitation assay was also performed. As shown, Bcl-2 ubiquitination was suppressed after ectopic expression of *PKCα* whereas depletion of *PKCα* led to enhanced ubiquitination (Figure 5C). Moreover, NDRG1 could reverse the suppression of Bcl-2 ubiquitination induced by PKCα (Figure 5D). Therefore, we speculated that NDRG1 might specially block the effect of PKCα. Subsequently, western blots and RT-PCR were performed in HCT116 *NDRG1* derivates to detect potential alterations of PKCα. Unfortunately, no statistic differences were detected in either way (Figure 5E and Supplementary Figure 2F). Co-immunoprecipitation assay was finally performed to test potential direct interaction between NDRG1 and PKCα. As shown, NDRG1 directly binded PKCα in HCT116 and SW620 cells (Figure 5F). These results indicated that NDRG1 might inhibit PKCα function via direct binding. As consequences, ubiquitination of Bcl-2 was enhanced and CRC cells were more sensitive to apoptosis induced by oxaliplatin.

### NDRG1 inhibited tumor growth and promoted apoptosis *in vivo*

**Figure 6:**
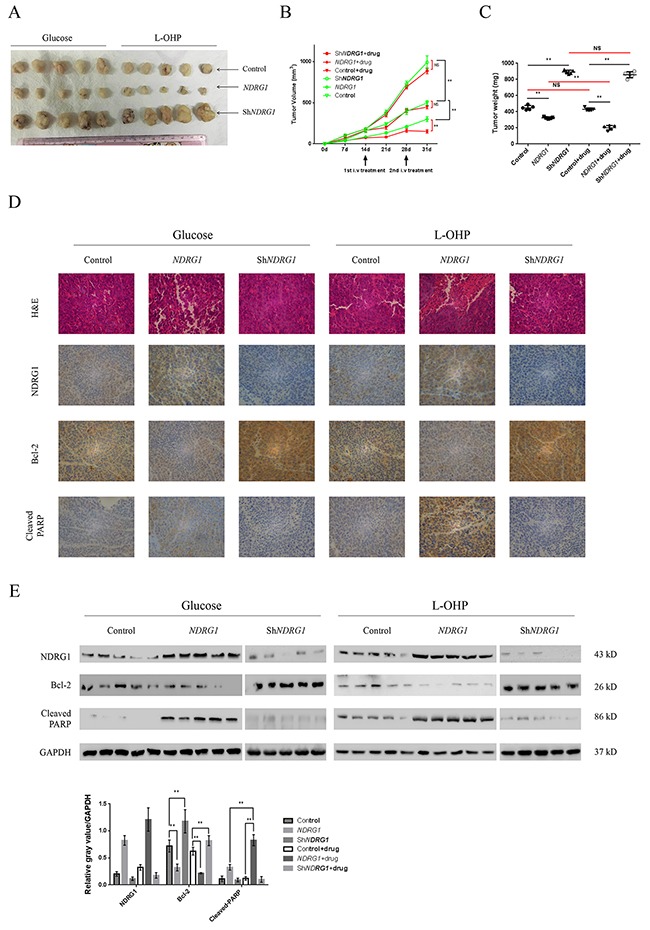
NDRG1 inhibited tumor growth and promoted apoptosis *in vivo* **(A)** Representative image of xenografts formed by derivates of HCT116 CRC cells. The xenograft tumors were from control (glucose) or treated (L-OHP) group as indicated. **(B, C)** Quantitative analysis of tumor volume (B) and tumor weight (C). Arrows indicated two-time infusion of oxaliplatin from the tail vein. Red line indicated comparison between drug-treated and -untreated groups. **(D)** Immunohistochemical staining of NDRG1, Bcl-2, and cleaved-PARP in tumor xenografts with hematoxylin counterstaining. Magnification, ×200. **(E)** Western blots showing protein levels of NDRG1, Bcl-2 and cleaved-PARP in xenograft tumor samples from HCT116-control, -*NDRG1* and –sh*NDRG1* groups with or without drug treatment. Bars indicated SD. NS, not significant, *, *P* < 0.05, **, *P* < 0.01.

Based on our *in vitro* findings, xenograft model was established to examine the *in vivo* effect of NDRG1 by injecting HCT116*NDRG1* or -sh*NDRG1* cells into nude mice subcutaneously (Figure 6A). Oxaliplatin (5mg/kg in 5% glucose solution) was infused from the tail vein to simulate the neoadjuvant chemotherapy in human rectal cancer patients. After the infusion, the growth of *NDRG1* xenografts was significantly suppressed comparing with those from mice receiving glucose (Figure 6B, 6C). Moreover, xenograft tumors formed by control and sh*NDRG1* cells still underwent rapid growth even after drug treatment (Figure 6B, 6C). In addition, the levels of NDRG1 and Bcl-2 in xenografts were detected by IHC (Figure 6D and Supplementary Figure 2G) and western blots (Figure 6E). As shown, expression of *NDRG1* was always associated with decreased Bcl-2 and enhanced apoptosis. Accordingly, depletion of *NDRG1* led to accumulation of Bcl-2 and impaired apoptosis. These data were consistent with our *in vitro* observations and suggested that the NDRG1 suppressed tumor growth and promoted apoptosis *in vivo*.

## DISCUSSION

Even after recommended by guidelines, the necessity of including oxaliplatin into neoadjuvant chemotherapy has been controversial. Plenty of clinical trials concerning rectal cancer patients dominated by T4 and advanced T3 cases given sequential oxaliplatin-containing preoperative chemotherapy demonstrated high tumor response rates, prolonged overall survival, and acceptable toxicity [4, 5, 7]. Yet other researchers [34, 35] didn't observe similar promising results, suggesting that rectal cancer might be biologically heterogeneous with subgroups eligible for further individualization of therapy. Our investigation showed that NDRG1 was positively correlated with the downstaging rates and prognosis of LARC patients receiving neoadjuvant chemotherapy. Moreover, substantial apoptosis in cancer tissues from downstaging patients was detected by a pathscan array. These finding inspired us to focus on the role of NDRG1 in apoptosis.

Apoptosis is one of the major mechanisms of cell death in response to chemotherapy [36]. Alterations in susceptibility to apoptosis not only contribute to tumor development but also can grant resistance to anticancer therapies. In our study, elevated levels of P53, cleaved-caspase-3 and cleaved-PARP were revealed by pathscan array. The P53 tumor suppressor protein can induce expression of numerous pro-apoptotic gene products which can activate intrinsic or extrinsic apoptotic pathways [37]. Current evidence points toward P53 functioning through transcriptional activation of pro-apoptotic target genes that contain *TP53* gene binding sites within their regulatory regions [38]. Of the several genes to which P53 can directly target, NDRG1 has been considered to be necessary for P53-dependent apoptosis in several types of cancer including colorectal cancer [24]. NDRG1 is a potent metastasis suppressor in multiple tumor-types [39]. Plenty of studies manifest that over-expression of *NDRG1* is able to inhibit the invasion and metastasis of CRC [40, 41]. In contrast, some studies have demonstrated that *NDRG1* promotes cell migration and invasion in certain type of carcinoma [42]. Hence, the role of *NDRG1* in the formation, development and treatment of cancers may depend on tissue-specific molecular profiles and remains to be clarified. We showed that *NDRG1* increased sensitivity of CRC cells to oxaliplatin-triggered apoptosis. In line with this, knockdown of *NDRG1* led to impaired response to chemotherapy. Moreover, in *TP53* defective cancer cells, the ectopic *NDRG1* expression could restore the response of CRC cells to oxaliplatin treatment. These results indicate that one of the major mechanisms by which P53 activate apoptotic pathway is targeting and activating *NDRG1*. Once *NDRG1* is activated, it is sufficient to maintain the response of cancer cells to drug-induced apoptosis.

Dysregulation or blockade of apoptosis in cancer cells represents a potential mechanism of resistance to anticancer therapies [43, 44]. Given the promoting activity of NDRG1 in apoptosis, we speculated that there might be interactions between NDRG1 and other apoptosis regulating proteins. As expected, the level of Bcl-2, a potent inhibitor of intrinsic apoptotic pathway [37], was decreased upon the expression of NDRG1. Over-expression of anti-apoptotic Bcl-2 family members in cancer has been associated with chemotherapy resistance in various human cancers, and preclinical studies have shown that agents targeting anti-apoptotic Bcl-2 family members have preclinical activity as single reagent and in combination with other anticancer drugs [45]. Thus, NDRG1 may exert its pro-apoptotic function through down-regulating Bcl-2. It was previously reported that the posttranscriptional modulation of Bcl-2, mainly through ubiquitination and phosphorylation, was responsible for its stability [46, 47]. Bcl-2 was shown ubiquitinated and rapidly degraded in apoptosis trigged by TNF-α [48] and UV exposure [49]. Furthermore, attenuation of this ubiquitination process contributed to chemo-resistance in cancer cells. Therefore, the process of regulating Bcl-2 stability seems to be crucial in sensitizing cancer cells to chemotherapeutics. In our subsequent investigation, we focused on the cellular degradative machinery for Bcl-2. Lysosomal and proteasomal pathways were both explored. We found that MG132, a proteasome inhibitor, led to accumulation of Bcl-2 protein in *NDRG1*-transfected cells, which suggested the degradation of Bcl-2 induced by NDRG1 was blocked. Thus, Bcl-2 degraded through proteasome pathway. Moreover, immunoprecipitation assay provided solid evidence that NDRG1 enhanced Bcl-2 ubiquitination and subsequent proteasomal degradation. In the absence of Bcl-2, CRC cells were more sensitive to oxaliplatin-triggered apoptosis.

It has been reported that that PKC or Erk1/2 prolonged half life of Bcl-2 via phosphorylation of several sites in Bcl-2 protein [50]. Among several isoforms, PKCα was proved to suppress Bcl-2 ubiquitination and increased its stability. We then investigated several potential mechanisms and finally discovered that NDRG1 might suppress PKCα function via direct binding. Through this, NDRG1 could enhance Bcl-2 ubiquitination and promote apoptosis. However, the detailed molecular mechanisms through which NDRG1 affects PKCα remains to be clarified in further studies.

The present study highlights the pivotal role of NDRG1 in regulating apoptosis of CRC. In summary, through interacting with PKCα, NDRG1 enhances the degradation of Bcl-2 and promotes apoptosis in CRC. In the presence of NDRG1, oxaliplatin-based chemotherapy can yield great killing activity, resulting in higher downstaging rates and better prognosis of LARC patients. As far as we are concerned, our study is the first demonstration that rectal cancer can be stratified by *NDRG1* status in terms of the response to chemotherapy. These findings provide a novel predictive biomarker for the treatment of CRC. However, this conclusion needs to be confirmed in further clinical trial.

## MATERIALS AND METHODS

### Ethics statement

Investigation has been conducted in accordance with the ethical standards and according to the Declaration of Helsinki and according to national and international guidelines and has been approved by the Medical Ethics Committee of Ruijin Hospital. Informed consent for medical research and publication has been obtained from all patients.

### Patients and treatment strategy

This study was performed to evaluate the clinical outcome of patients with locally advanced rectal cancer treated with neoadjuvant chemotherapy. Patients enrollment criteria were as follows: age between 18 and 70 years; primary rectal cancer; histological diagnosis of adenocarcinoma; TNM stage ≥ T3 or positive regional lymph nodes revealed by endoscopic ultrasonography or magnetic resonance images (MRI); distance to anal verge ≤ 10 cm; Eastern Cooperative Oncology Group (ECOG) performance status score ≤ 2; no history of previous chemotherapy. All the patients were given a clinical stage either by endoscopic ultrasonography (EUS) or magnetic resonance imaging (MRI) prior to treatment. Oxaliplatin-based chemotherapy was then delivered to all the patients for two cycles before surgery. Patients receiving FOLFOX regimen (oxaliplatin 85 mg/m^2^ on day 1, leucovorin 200 mg/m^2^ on day 1, 5-Fu 400 mg/m^2^ intravenous infusion on day 1, then 600 mg/m^2^/day continuous infusion for 2 days) or XELOX regimen (oxaliplatin 100 mg/m^2^ on day 1, capecitabine 1,000 mg/m^2^ bid on days 1–14) were all included. Surgery was scheduled within 6-8 weeks after the completion of chemotherapy, and standard TME was then performed. For each patient, the resected specimen was examined by two independent pathologists for pathological staging. The pathological stage was compared with the pre-chemotherapy stage. Complete regression (CR) was defined as no cancer cells in the rectal wall or mesorectum (ypT0N0). Downstaging was defined as achieving some tumorous and lymphatic regression including CR. No-downstaging was defined as no difference between pre-chemotherapy stage and final pathological stage or TNM stage upgrading during preoperative chemotherapy. All patients were routinely followed. Sixteen samples were selected by simple randomization conducted with SAS software. Protein and RNA for further research were obtained from specimen of preoperative colonoscopy biopsy and surgical resection.

### Reagents and anitbodies

The Pathscan Sampler Kit (#7851) and Apoptosis Antibody Sampler Kit (#9930) including anti-cleaved PARP, anti-cleaved caspase-3, anti-cleaved caspase-8, and anti-cleaved caspase-9 antibodies were purchased from Cell Signaling Technology (Beverly, MA, USA). The In Situ Cell Death Detection Kit (11684795910) was obtained from Roche (Indianapolis, IN, USA). Anti-P53 (#2524), anti-Bcl-2 (#15071) antibodies were also obtained from Cell Signaling Technology. Anti-NDRG1 antibody (WH0010397M3) was from Sigma Aldrich (St. Louis, MO, USA). Anti-PKCα (#2056) antibody was obtained from Cell Signaling Technology. Anti-PKCβ (2099) antibody was obtained from Santa Cruz Biotechnology (Santa Cruz, CA, USA). Oxaliplatin (3422) was provided by Sanofi (Paris, France). The PKC inhibitor GO6976 (S7119) was obtained from Selleck (Houston, TX, USA).

### Pathscan array

Conducting of pathscan array was strictly followed the manufacturer's protocol. In brief, the glass slides containing detecting antibody matrix was prepared and assembled. Then the blocking buffer was added in each well and incubated in room temperature for 15 minutes. After washing, a total protein of 30μg was added in each well and incubated at 4°C overnight on an orbital shaker. Then the slide was washed with PBS and placed on a plastic dish. The images were captured using an Oddessy fluorescent imaging system (LI-COR Biotechnology, Lincoln, NE, USA).

### Cell culture and transfections

Human colorectal cancer cells HCT116, SW620 and HT29 were from American Type Culture Collection (ATCC). Standard growth media for HCT116 and HT29 was McCoy's 5A (Invitrogen, Carlsbad, CA, USA) supplemented with 10% fetal bovine serum (FBS, HyClone, Logan, UT, USA). SW620 was cultured in Leibovitz's L-15 medium (Gibco) with 10% FBS. All cells were maintained in a 37°C incubator with 5% CO_2_. For transient transfection, cells were transfected with pcDNA3.1 (over-expression) or pLKO (knockdown) plasmids encoding target sequences. For establishment of stable cell Lines, the stable clones were selected by puromycin (5 μg/mL) for 2 weeks. Sequences or hairpin sequences used for construction were listed in Table 3. All constructs were confirmed by sequencing.

**Table 3:** Sequences for construction of plasmids

Name	Oligonucleotides (5’–3’)	
	Forward primer	Reverse primer
**NDRG1**	GTGCAGAAGGGACTAGGCAG	GGGCACCCACGTAATAGACC
**Bcl-2**	CGTGATTGAAGACACCCCCT	CAGCCTGCAGCTTTGTTTCAT
**Bcl-2 mutant**	CGCTGGGAGAACAGGGTACTATAAGCTGTCGCAGAG	CTCTGCGACAGCTTATAGTACCCTGTTCTCCCAGCG
**PKCα**	GCGGAGGCAAGAGGTGG	AGGATTCACTTCCCACTGCG
**PKCβ**	CAGCTGGGCGAGTGACAG	CCAGGCTCAACGATGGAGTT
	**Hairpin sequences**	
**Sh-NDRG1**	CCGGGCCTACATCCTAACTCGATTTCTCGAGAAATCGAGTTAGGATGTAGGCTTTTTG	
**Sh- PKCα**	CCGGCCCGTCTTAACACCACCTGATCTCGAGATCAGGTGGTGTTAAGACGGGTTTTT	
**Sh- PKCβ**	CCGGCCGGATGAAACTGACCGATTTCTCGAGAAATCGGTCAGTTTCATCCGGTTTTT	

### Western blot and immunoprecipitation

For western blot analysis, cells were lysed in RIPA buffer (50 mM Tris-HCl, pH 6.8, 8 M urea, 5% β-mercaptoethanol, 2% SDS, and 1 × protease inhibitor mixture). The total cell lysates was sonicated and then centrifuged at 14,000g for 5 min at 4 °C to remove cell debris. The supernates were then separated in 12.5% SDS-PAGE. Protein levels were revealed by enhanced chemiluminescence (ECL) method. For Bcl-2 immunoprecipitation assay, protein extracts prepared in lysis buffer (1% CHAPSO, 30 mM Tris-HCl, pH 8.0, 150 mM NaCl, 5 mM EDTA, and 1 × protease inhibitor mixture) were firstly pre-cleared to reduce non-specific binding of proteins to sepharose beads. The supernatants was incubated with Bcl-2 antibody overnight and then with protein A/G agarose for 4 hours at 4 °C. After washing five times with cold PBS, the immunoprecipitates was boiled and subjected to western blot analysis with anti-ubiquitin antibody. For detecting NDRG1/PKC binding, 5×10^6^ cells were harvested with immunoprecipitation lysis buffer (20 mM Tris-HCl, pH 7.6; 150 mM NaCl; 1 mM EDTA; 0.5% NP-40; 10% glycerol; 1 mM PMSF; protease inhibitor cocktail). After sonication, the lysates were centrifuged (14,000g, 15min) at 4°C. Immunoprecipitation was performed using anti-NDRG1 antibody. After adding protein A/G agarose, equal amounts of lysates were incubated with NDRG1 antibody or IgG for 16h. Subsequently, immunoprecipitates were washed 3 times with RIPA buffer. All incubations and washes were performed at 4°C with gentle rotation. Proteins were eluted from beads for western blots.

### Reverse transcription-polymerase chain reaction (RT-PCR)

RT-PCR was performed as we described previously [41]. The involving primers were as follows: NDRG1 forward: 5’-CTCCTGCAAGAGTTTGATGTCC-3’ and reverse: 5’-TCATGCCGATGTCATGGTAGG-3’. PKCα forward: 5’-GTCCACAAGAGGTGCCATGAA-3’ and reverse: 5’-AAGGTGGGGCTTCCGTAAGT-3’.

### Flow cytometry analysis

For flow cytometry analysis, both floating and attached cells were collected, after washing twice with ice-cold PBS, cells were resuspended in 400 μl binding buffer (10 mM HEPES, 140 mM NaCl, and 2.5 mM CaCl_2_, pH 7.4) and stained with annexin V-enhanced green fluorescent protein/propidium iodide (PI), using an annexin V-enhanced green fluorescent protein apoptosis detection kit (BD science, UK). After incubation for 15 minutes, the cells were collected and analyzed in a FACScanflow cytometry analyzer (Beckman, Brea, CA, USA) following the manufacturer's protocol. Both PI- and annexin V-negative cells (quadrant 4) were intact cells, PI-negative and annexin V-positive cells were considered as early apoptotic (quadrant 3) cells, both PI- and annexin V-positive (quadrant 2) cells were late apoptotic cells, PI-positive and annexin V-negative cells were considered to be mechanically injured (quadrant 1) during the experiment.

### *In vivo* studies

Mice were maintained at the animal experiment center of Ruijin Hospital under standard conditions following institutional guidelines. All procedures were approved by the Institute Ethical Committee for animal use. A total of 10×10^6^ HCT116*NDRG1* or HCT116sh*NDRG1* cells along with control cells were injected subcutaneously into nude mice (male BALB/c nu/nu nude mice, 4-week-old, 5 per group). To assess the effect of oxaliplatin on different *NDRG1* derivates *in vivo*, mice were divided into two groups receiving oxaliplatin treatment or glucose as a control. Treatments were started from day 14. The treatment schedule consisted of 2 intravenous infusions, every 2 weeks, 5 mg/kg injection of oxaliplatin from tail vein each time. The drug dose was decided on the basis of literature data and results of our preliminary experiment showing that this dose was generally well tolerated whereas higher dosages were associated with death of mice. Tumor size was measured every 7 days using calipers and tumor volume (V) was determined by measuring the length and width of the tumor and using the formula V = (width^2^ × length) / 2.

### Immunohistochemical staining

Immunohistochemical (IHC) staining was performed on paraffin-embedded tissue sections. The tissue sections were deparaffinized, rehydrated, and microwaved-heated in sodium citrate buffer (10mmol/L, pH6.0) for antigen retrieval. Then, the slides were incubated with primary antibody (anti-NDRG1, anti-Bcl-2, or anti-cleaved PARP). Counterstaining was performed using hematoxylin. The scoring was carried out by two independent pathologists blinded to the clinical characteristics of the patients according to proportion of cell staining (0 = 0%, 1 = ≤ 25%, 2 = 26% to 50%, 3 = 51% to 75%, 4 = > 75% positive cells) and the staining intensity (0 = no staining, 1 = weak, 2 = moderate, 3 = strong). Scores of cell staining and staining intensity were multiplied. An overall score of ≤ 7 was defined as negative, while a score >7 was defined as positive.

### Statistics

SPSS software (version 19.0) was utilized for statistical analysis. Differences between continuous variables (protein levels and RNA levels) were compared using unpaired *t* test. Relationship between NDRG1 expression and clinicopathologic variables were measured by Pearson Chi-square (χ2) test. *R* values were determined by Spearman correlation analysis. Overall survival was assessed by Kaplan-Meier method, and difference between survival curves was determined by using the log-rank test. Growing curves of xenograft tumors from different NDRG1 derivates were compared with two-way analysis of variance (ANOVA) method. Differences with a *P* value < 0.05 were considered as statistically significant.

## SUPPLEMENTARY MATERIALS FIGURES AND TABLES





## Abbreviations

CRC: colorectal cancer
NDRG1: N-myc downstream regulated gene-1
RT-PCR: reverse transcription-polymerase chain reaction
CRT: chemoradiation therapy
TME: total mesorectal excision
LARC: locally advanced rectal cancer.
