# Supplementary material for: N-myc downstream-regulated gene 1 promotes oxaliplatin-triggered apoptosis in colorectal cancer cells via enhancing the ubiquitination of Bcl-2
Source: Oncotarget. 2017 May 9;8(29):47709–24. doi: 10.18632/oncotarget.17711 (PMC5564599; doi:10.18632/oncotarget.17711)
Supplement: Supplementary file 1 [file oncotarget-08-47709-s001.pdf]

N-myc downstream-regulated gene 1 promotes oxaliplatin-triggered apoptosis in colorectal cancer cells via enhancing the ubiquitination of Bcl-2

SUPPLEMENTARY FIGURES AND TABLE

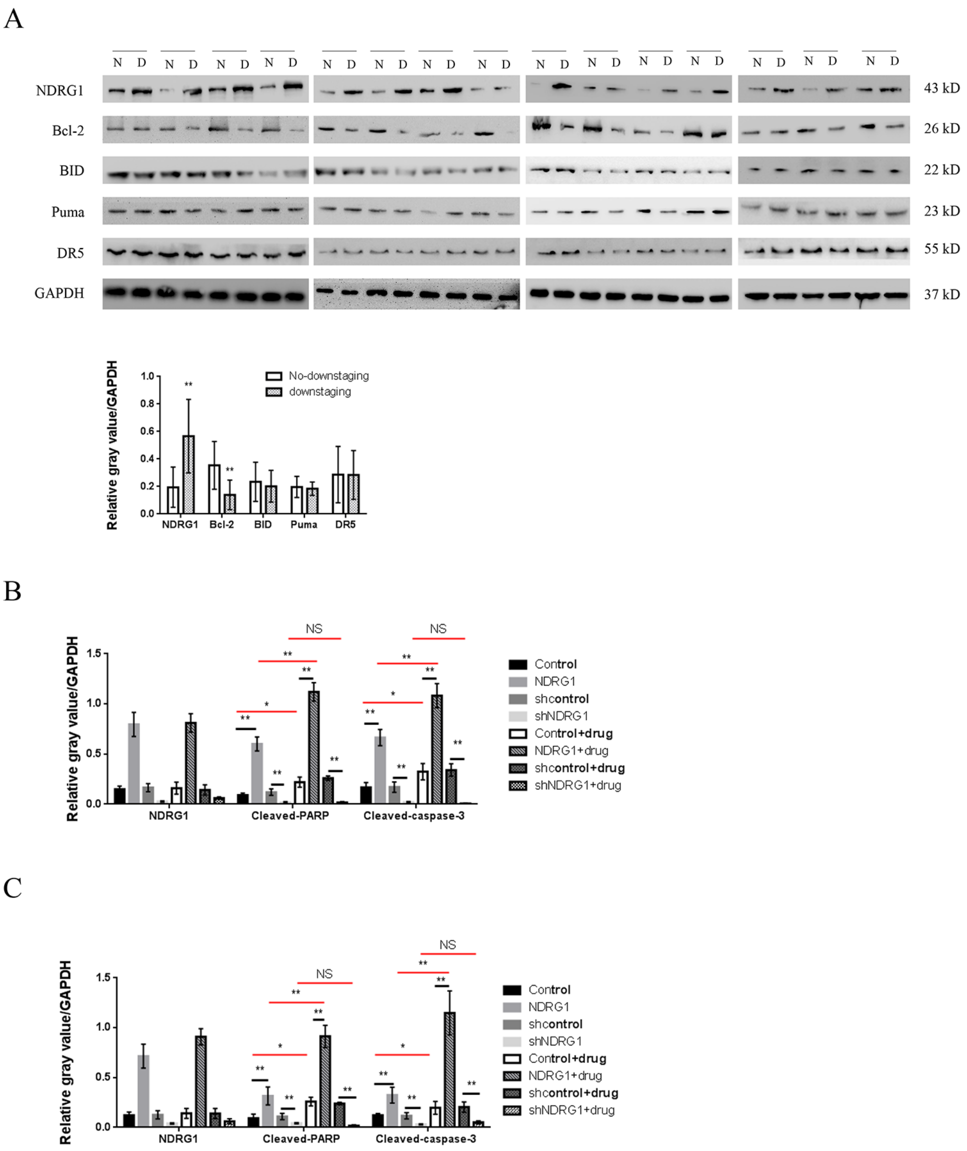

Supplementary Figure 1: (A) Western blots showing protein levels of several apoptosis regulating factors namely NDRG1, Bcl-2, Puma, BID, and death receptor 5 (DR5) in selected patients from no-downstaging and downstaging group respectively. **(B, C)** Quantitative analysis of protein levels showed in Figure. 2C (B) and 2D (C). Red line indicated comparison between drug-treated and -untreated groups. Data were from three independent experiments. Bars indicated SD. NS, not significant, *, $P < 0.05$, **, $P < 0.01$.

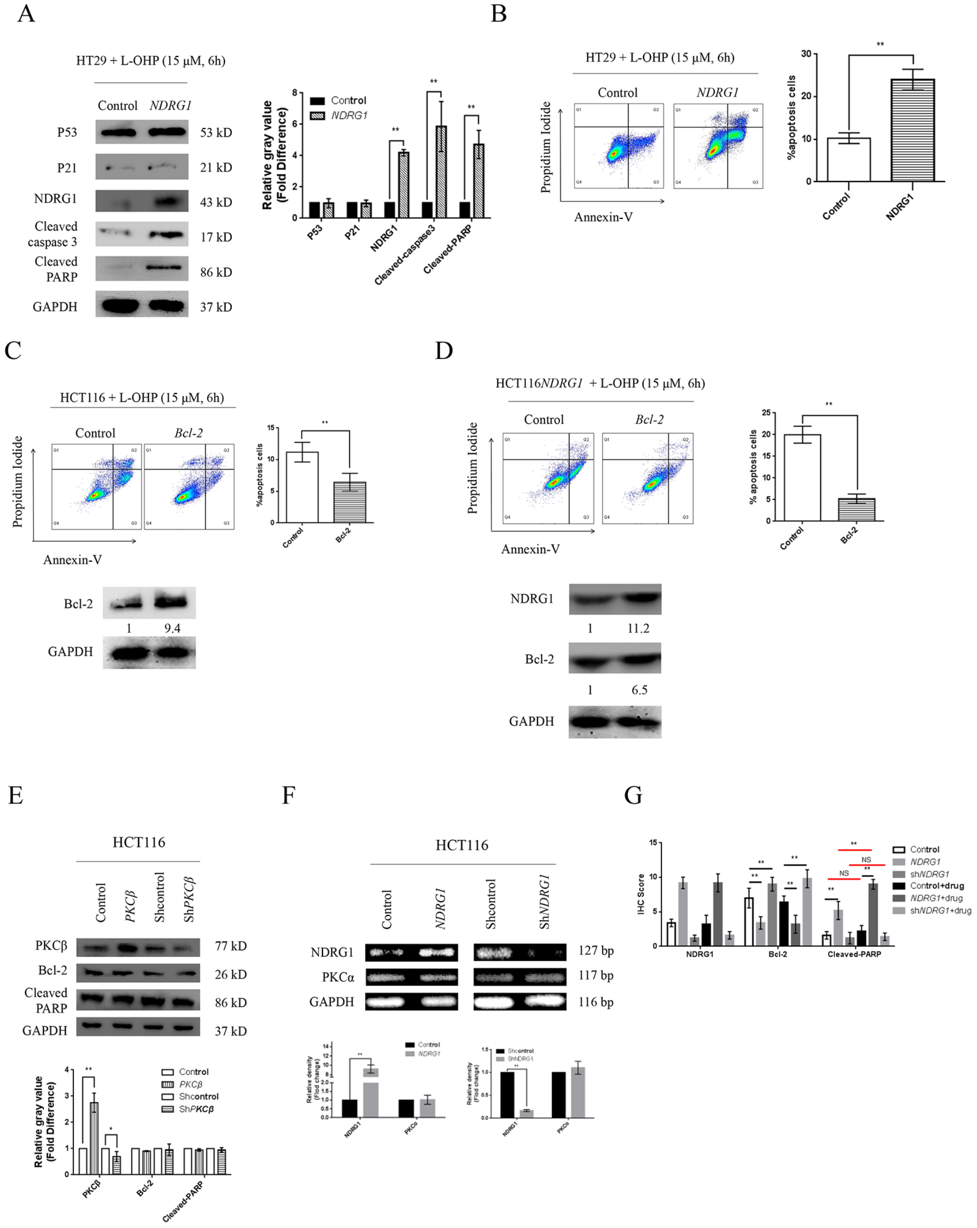

Supplementary Figure 2: (A) *NDRG1* restored the response of HT29 cells to oxaliplatin. After 6 hours incubation with oxaliplatin (15μM), apoptosis in HT29*NDRG1* cells and control cells were analyzed with western blots. Low level of P21 was an evidence of defective *TP53* gene in HT29 cells. **(B)** Apoptotic cell numbers in HT29 *NDRG1* derivates were analyzed by flow cytometry assay with Annexin V/PI. **(C, D)** Large amounts (10μg) of *Bcl-2* constructs or control vector were transfected into HCT116 cells (C) or HCT116-*NDRG1* (D) cells. Transfects were then cultured in medium containing oxaliplatin (15μM). Apoptosis were analyzed with flow cytometry assay. The successful transfection of *Bcl-2* and *NDRG1* were confirmed by western blots. Densitometry showed relative protein expression normalized for GAPDH. **(E)** *PKCβ* status had no effect on the stablity of Bcl-2 or apoptosis. HCT116 cells were transfected with *PKCβ*, sh*PKCβ* or control vector. Protein level of Bcl-2 was then detected with western blot. **(F)** NDRG1 was unable to modulate mRNA level of *PKCα*. After transfection of *NDRG1* constructs or control vector (10μg), total RNA was extracted and subjected to RT-PCR. **(G)** Quantitative analysis of IHC scores of Figure. 6D. Red line indicated comparison between drug-treated and -untreated groups. Histograms showed mean values from three independent experiments; bars indicated SD; NS, not significant, *, $P < 0.05$, **, $P < 0.01$.

Supplementary Table 1: Detailed information of patients.

See Supplementary File 1
