# Supplementary material for: N-myc downstream-regulated gene 1 promotes oxaliplatin-triggered apoptosis in colorectal cancer cells via enhancing the ubiquitination of Bcl-2
Source: Oncotarget. 2017 May 9;8(29):47709–24. doi: 10.18632/oncotarget.17711 (PMC5564599; doi:10.18632/oncotarget.17711)
Supplement: Supplementary file 2 [file oncotarget-08-47709-s002.docx]

Supplementary Table 1: Detailed information of patients.

| Case number | Clinical TNM stage(EUS/MRI) | Pathologic TNM stage | Stage | NDRG1 status | Conclusion |
| --- | --- | --- | --- | --- | --- |
| 1 | T3N2a | T2N1 | III | N | PR |
| 2 | T2N2a | T2N1 | III | N | PR |
| 3 | T3N1 | T2N1 | III | N | PR |
| 4 | T3N2a | T2N1 | III | N | PR |
| 5 | T3N2a | T2N1 | III | P | PR |
| 6 | T3N1 | T2N1 | III | P | PR |
| 7 | T4N2a | T2N1 | III | P | PR |
| 8 | T2N1 | T2N1 | III | P | SD |
| 9 | T3N1 | T2N1 | III | P | PR |
| 10 | T3N2a | T2N1 | III | P | PR |
| 11 | T3N2b | T2N1 | III | P | PR |
| 12 | T2N1 | T2N1 | III | P | SD |
| 13 | T3N1 | T2N1 | III | P | PR |
| 14 | T3N1 | T2N1 | III | P | PR |
| 15 | T2N1 | T2N1 | III | P | SD |
| 16 | T3N1 | T2N1 | III | P | PR |
| 17 | T3N2a | T2N1 | III | P | PR |
| 18 | T3N1 | T2N1 | III | P | PR |
| 19 | T4N1 | T2N1 | III | P | PR |
| 20 | T3N1 | T2N1 | III | P | PR |
| 21 | T4N2a | T2N1 | III | P | PR |
| 22 | T3N1 | T2N1 | III | P | PR |
| 23 | T3N1 | T2N1 | III | P | PR |
| 24 | T3N1 | T2N1 | III | P | PR |
| 25 | T3N2b | T2N1 | III | P | PR |
| 26 | T2N1 | T2N1 | III | P | SD |
| 27 | T3N1 | T3N0 | II | N | PR |
| 28 | T3N0 | T3N0 | II | N | SD |
| 29 | T3N1 | T3N0 | II | P | PR |
| 30 | T3N1 | T3N0 | II | P | PR |
| 31 | T3N0 | T3N0 | II | P | SD |
| 32 | T3N0 | T3N0 | II | P | SD |
| 33 | T3N1 | T3N0 | II | P | PR |
| 34 | T3N1 | T3N0 | II | P | PR |
| 35 | T3N1 | T3N0 | II | P | PR |
| 36 | T3N1 | T3N0 | II | P | PR |
| 37 | T3N2a | T3N0 | II | P | PR |
| 38 | T3N1 | T3N0 | II | P | PR |
| 39 | T4N2a | T3N1 | III | N | PR |
| 40 | T3N1 | T3N1 | III | N | SD |
| 41 | T4N2a | T3N1 | III | N | PR |
| 42 | T3N0 | T3N1 | III | N | PD |
| 43 | T3N1 | T3N1 | III | N | SD |
| 44 | T4N2a | T3N1 | III | N | PR |
| 45 | T3N1 | T3N1 | III | N | SD |
| 46 | T3N1 | T3N1 | III | N | SD |
| 47 | T4N2a | T3N1 | III | N | PR |
| 48 | T4N2a | T3N1 | III | N | PR |
| 49 | T3N1 | T3N1 | III | N | SD |
| 50 | T3N1 | T3N1 | III | N | SD |
| 51 | T3N1 | T3N1 | III | N | SD |
| 52 | T3N1 | T3N1 | III | N | SD |
| 53 | T4N2a | T3N1 | III | N | PR |
| 54 | T4N2a | T3N1 | III | N | PR |
| 55 | T4N2a | T3N1 | III | N | PR |
| 56 | T3N2b | T3N1 | III | N | PR |
| 57 | T3N2b | T3N1 | III | N | PR |
| 58 | T4N2a | T3N1 | III | N | PR |
| 59 | T3N0 | T3N1 | III | P | PD |
| 60 | T3N0 | T3N1 | III | P | PD |
| 61 | T3N1 | T3N1 | III | P | SD |
| 62 | T3N0 | T3N1 | III | P | PD |
| 63 | T3N1 | T3N1 | III | P | SD |
| 64 | T3N0 | T3N1 | III | P | PD |
| 65 | T3N1 | T3N1 | III | P | SD |
| 66 | T3N1 | T3N1 | III | P | SD |
| 67 | T3N1 | T3N1 | III | P | SD |
| 68 | T3N1 | T3N1 | III | P | SD |
| 69 | T4N2a | T3N1 | III | P | PR |
| 70 | T3N0 | T3N1 | III | P | PD |
| 71 | T3N0 | T3N2a | III | N | PD |
| 72 | T3N1 | T3N2a | III | N | PD |
| 73 | T3N2a | T3N2a | III | N | SD |
| 74 | T3N2a | T3N2a | III | N | SD |
| 75 | T3N1 | T3N0 | II | N | PR |
| 76 | T4N1 | T3N0 | II | P | PR |
| 77 | T3N1 | T3N0 | II | P | PR |
| 78 | T3N1 | T3N0 | II | P | PR |
| 79 | T3N1 | T3N0 | II | P | PR |
| 80 | T4N1 | T3N0 | II | P | PR |
| 81 | T3N1 | T3N0 | II | P | PR |
| 82 | T3N1 | T3N0 | II | P | PR |
| 83 | T3N1 | T3N0 | II | P | PR |
| 84 | T4N1 | T3N0 | II | P | PR |
| 85 | T3N1 | T3N0 | II | P | PR |
| 86 | T3N1 | T3N0 | II | P | PR |
| 87 | T4N0 | T4N1 | III | N | PD |
| 88 | T4N0 | T4N1 | III | N | PD |
| 89 | T4N1 | T4N1 | III | N | SD |
| 90 | T4N0 | T4N1 | III | N | PD |
| 91 | T4N1 | T4N1 | III | N | SD |
| 92 | T4N1 | T4N1 | III | P | SD |
| 93 | T4N1 | T4N2a | III | N | PD |
| 94 | T4N1 | T4N2a | III | N | PD |
| 95 | T4N1 | T4N0 | II | N | PR |
| 96 | T4N1 | T4N0 | II | N | PR |
| 97 | T4N1 | T4N0 | II | P | PR |

N=Negative; P=Positive; PR=Partial response; PD=Progression; SD=Steady.
